# Supplementary material for: A Deep Learning Framework for Robust and Accurate Prediction of ncRNA-Protein Interactions Using Evolutionary Information
Source: Mol Ther Nucleic Acids. 2018 Mar 9;11:337–44. doi: 10.1016/j.omtn.2018.03.001 (PMC5992449; doi:10.1016/j.omtn.2018.03.001)
Supplement: Document S1. Figures S1 and S2 and Tables S1–S3 [file mmc1.pdf]

OMTN, Volume *11*

Supplemental Information

A Deep Learning Framework for Robust and Accurate Prediction of ncRNA-Protein Interactions Using Evolutionary Information

Hai-Cheng Yi, Zhu-Hong You, De-Shuang Huang, Xiao Li, Tong-Hai Jiang, and Li-Ping Li

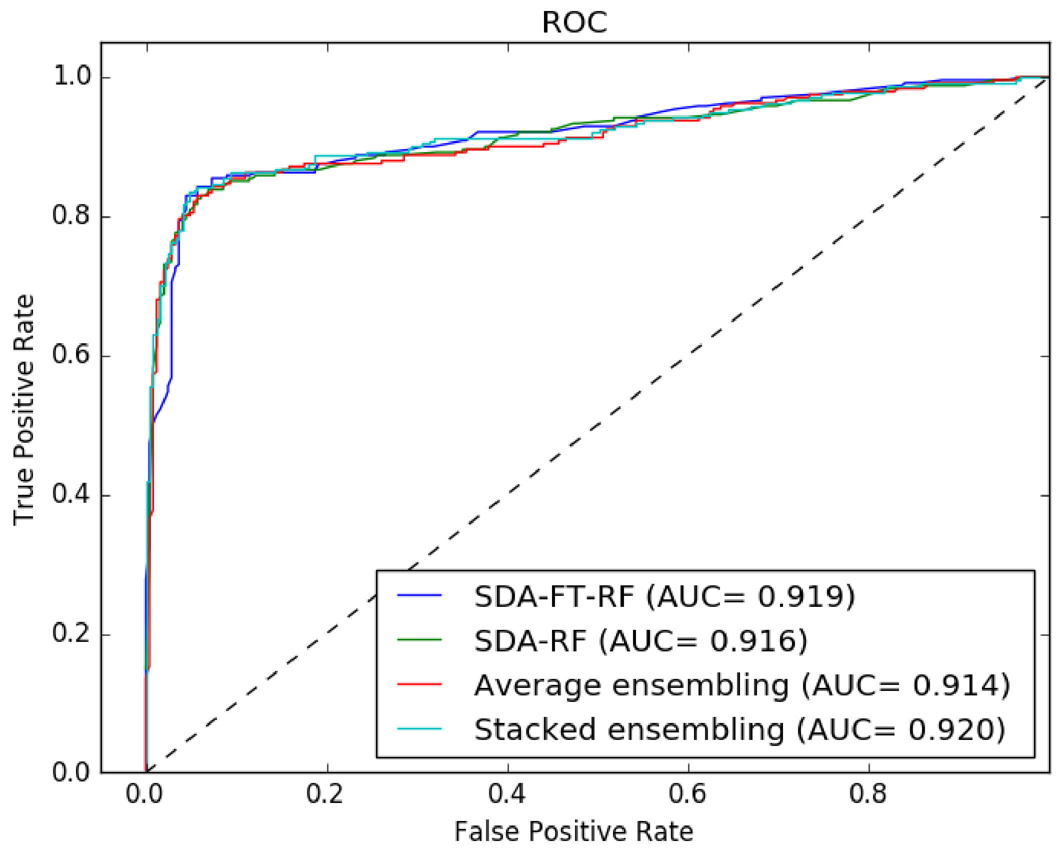

Figure S1: Performance on RPI488. Performance comparison between RPI-SAN, SDA-FT-RF, SDA-RF. Performance comparison between different assembling strategies.

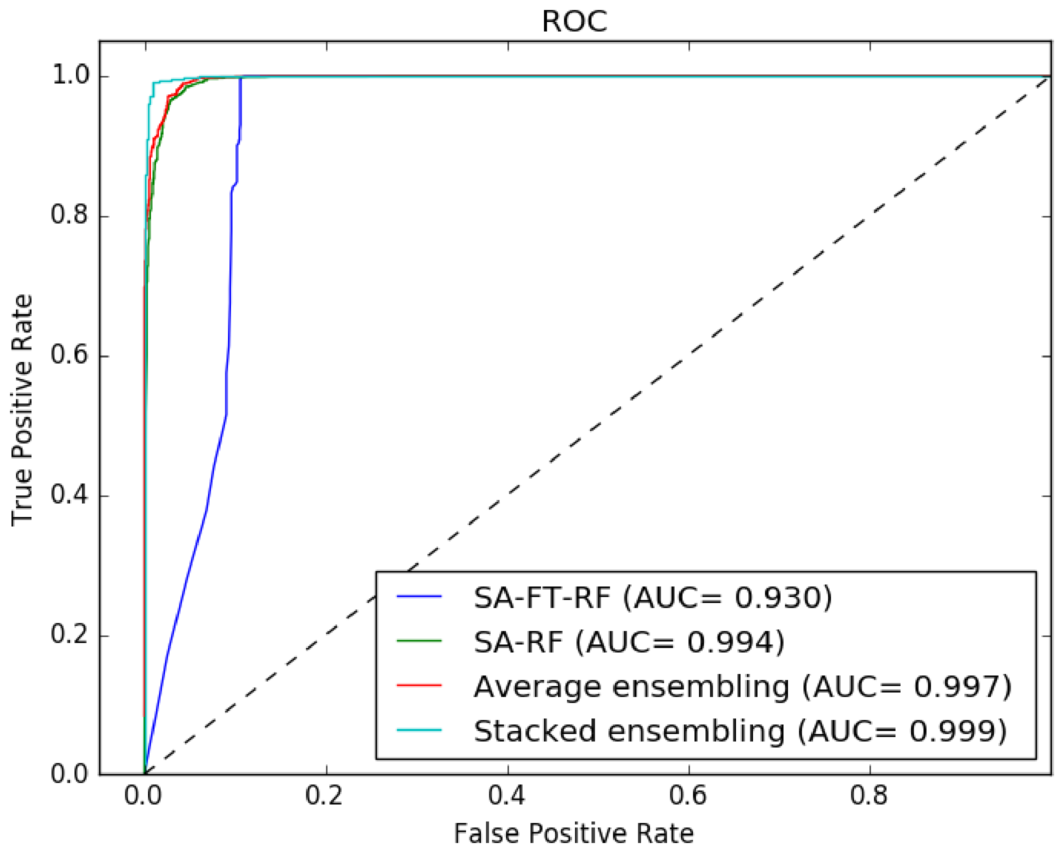

Figure S2: Performance on RPI1807. Performance comparison between RPI-SAN, SA-FT-RF, SA-RF. Performance comparison between different assembling strategies.

Table S1. K-mer sparse matrix based representation of RNA sequence.

	$R_1R_2R_3R_4$	$R_2R_3R_4R_5$	…	$R_{L-3}R_{L-2}R_{L-1}R_L$
**AAAA**	a_{11}	a_{12}	…	$a_{1,L-k+1}$
**AAAC**	a_{21}	a_{22}	…	$a_{2,L-k+1}$
**AACA**	a_{31}	a_{32}	…	$a_{3,L-k+1}$
**…**	…	…	…	…
**UUUU**	$a_{256,1}$	$a_{256,2}$	…	$a_{256,L-k+1}$

Table S2. The 5-fold cross-validation details on RPI2241 dataset.

Fold set	Accuracy(%)	Sensitivity(%)	Specificity(%)	Precision(%)	MCC(%)
**1**	90.50	86.08	96.82	84.06	81.63
**2**	90.50	85.38	97.96	82.91	81.89
**3**	91.65	85.80	99.99	83.14	84.46
**4**	90.84	87.53	95.46	86.11	82.00
**5**	90.38	86.06	96.60	84.03	81.36
**Average**	90.77±0.52	86.17±0.81	97.37±1.71	84.05±1.26	82.27±1.25

Table S3. Actual performance of individual predictors on RPI2241 dataset.

Predictors	Accuracy(%)	Sensitivity(%)	Specificity(%)	Precision(%)	MCC(%)
**SA-RF**	63.71	64.75	61.72	65.74	27.49
**SA-FT-RF**	90.52	**87.71**	94.78	**86.18**	81.56
**RPISeq-RF**	63.96	64.83	62.59	65.37	27.98
**lncPro**	65.4	65.9	64.0	66.9	31.0
**RPI-SAN**	**90.77**	86.17	**97.37**	84.05	**82.27**
