# Supplementary material for: A Deep Learning Framework for Robust and Accurate Prediction of ncRNA-Protein Interactions Using Evolutionary Information
Source: Mol Ther Nucleic Acids. 2018 Mar 9;11:337–44. doi: 10.1016/j.omtn.2018.03.001 (PMC5992449; doi:10.1016/j.omtn.2018.03.001)
Supplement: Document S2. Article plus Supplemental Information [file mmc2.pdf]

Molecular Therapy
Nucleic Acids
Original Article

A Deep Learning Framework for Robust and Accurate Prediction of ncRNA-Protein Interactions Using Evolutionary Information

Hai-Cheng Yi,[1,2,4] Zhu-Hong You,[1,4] De-Shuang Huang,[3] Xiao Li,[1] Tong-Hai Jiang,[1] and Li-Ping Li[1]

[1]Xinjiang Technical Institutes of Physics and Chemistry, Chinese Academy of Science, Urumqi 830011, China; [2]University of Chinese Academy of Sciences, Beijing 100049, China; [3]Institute of Machine Learning and Systems Biology, School of Electronics and Information Engineering, Tongji University, Shanghai, China

The interactions between non-coding RNAs (ncRNAs) and proteins play an important role in many biological processes, and their biological functions are primarily achieved by binding with a variety of proteins. High-throughput biological techniques are used to identify protein molecules bound with specific ncRNA, but they are usually expensive and time consuming. Deep learning provides a powerful solution to computationally predict RNA-protein interactions. In this work, we propose the RPI-SAN model by using the deep-learning stacked auto-encoder network to mine the hidden high-level features from RNA and protein sequences and feed them into a random forest (RF) model to predict ncRNA binding proteins. Stacked assembling is further used to improve the accuracy of the proposed method. Four benchmark datasets, including RPI2241, RPI488, RPI1807, and NPInter v2.0, were employed for the unbiased evaluation of five established prediction tools: RPI-Pred, IPMiner, RPISeq-RF, lncPro, and RPI-SAN. The experimental results show that our RPI-SAN model achieves much better performance than other methods, with accuracies of 90.77%, 89.7%, 96.1%, and 99.33%, respectively. It is anticipated that RPI-SAN can be used as an effective computational tool for future biomedical researches and can accurately predict the potential ncRNA-protein interacted pairs, which provides reliable guidance for biological research.

INTRODUCTION

In the *Human* genome, 74.7% of the sequence can be transcribed into RNA, but the total exon sequence of the mRNA is only 2.94%.[1–3] The remaining sequence information is output in the form of non-coding RNA (ncRNA), which can be divided into two types: constitutive and regulatory types.[4] The proportion of small molecule ncRNA in constitutive ncRNA and regulatory ncRNA is very small in non-coding sequences, and most of the non-coding sequences are transcribed into long ncRNA (lncRNA). Compared with mRNA, lncRNA is shorter in length, less in exon and two in focus, with an average abundance of about 1/10 of mRNA and a lower sequence conservation.[5–7] It has been found that lncRNA can participate in all aspects of gene expression regulation by interacting with proteins such as chromatin modification complexes and transcription factors, thus playing a fundamental role in a variety of important biological processes such as X chromosome inactivation (Xist[8] and Tsix[9]), gene imprinting (H19[10] and Air[11]), and developmental differentiation (HOTAIR[12] and TINCR[13]). Although the role of ncRNA-protein interactions (ncRPIs) in the regulation of gene expression has been doubtless, only a small number of ncRNA functions and mechanisms of action have been studied. Since ncRNA functions require the coordination of protein molecules, the identification of protein molecules bound with specific ncRNA has become the main approach to revealing the function and mechanism of ncRNA.

Large-scale RNA-binding proteins (RBPs) detection experiments based on biological methods have made many important advances,[14–16] such as RNAcompete,[17] HITS-CLIP,[18] and RNA-protein complex structure, which provide valuable information about the RNA-protein interactions (RPIs), while experimental methods are still time-consuming and overpriced (for example, it's high-cost to determine complex structure by way of experiment). These high-throughput technologies need much time for the abortive hand-tuning of putative binding sequences.[19] A lot of studies suggest that the sequences have enough information for predicting RPIs. The sequence-homology-based methods help to detect the binding domains of proteins and their possible functions,[20–24] but lack the ability to determine whether a given pair of RNA and protein can form the interaction well. There is an urgent need for an accurately computational approach to predicting RPIs.

In recent years, computational prediction of the interaction partner between proteins and RNAs has attracted a lot of research works.[15,25–35] Pancaldi et al.[36,37] trained a random forest (RF) and a

Received 10 October 2017; accepted 4 March 2018;
https://doi.org/10.1016/j.omtn.2018.03.001.

[4]These authors contributed equally to this work.

Correspondence: Zhu-Hong You, Xinjiang Technical Institutes of Physics and Chemistry, Chinese Academy of Science, Urumqi 830011, China.
E-mail: zhuhongyou@ms.xjb.ac.cn
Correspondence: De-Shuang Huang, Institute of Machine Learning and Systems Biology, School of Electronics and Information Engineering, Tongji University, Shanghai, China.
E-mail: dshuang@tongji.edu.cn

This is an open access article under the CC BY license (http://creativecommons.org/licenses/by/4.0/).

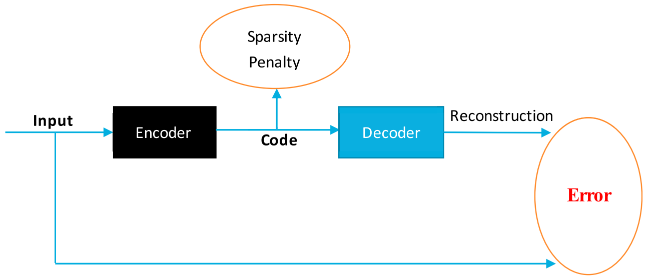

Figure 1. Prediction Performance Comparison Between SA-FT-RF, SA-RF, RPISeq-RF, Average Assembling, and Stacked Assembling on ncRNA-Protein Dataset RPI2241

support vector machine to classify whether the RNA-protein pair interact or not and used >100 different sources of features, which were extracted from genomic context,[38] structure, or localization. The RPISeq[21] was introduced by Muppirala et al.[39] They also applied RF and SVM classifiers by using simple 4-mer features of RNA and 3-mer features of proteins, respectively. Thereafter, lncPro[25] trained three types of physiochemical properties using Fisher linear discriminant. Zhou et al.[20] presented a new SVM based approach RPI-Pred by taking into consideration both sequences and structures information to predict ncRPIs. In the studies above, hand-crafted features of RNA-protein pairs are used in some methods,[38] which may change the real distribution back of the data and need strong domain knowledge. Other researchers extracted lowly discriminated features from noisy sequences, though they mainly got information from extracted sequences.[21,25,26] General machine learning methods might not mine the hidden regular pattern from these noises well. Thus, efficient features and advanced models play an important role in RPI's computational prediction.[40–43]

In this study, we propose a powerful solution for these challenges. It's a sequence-based approach to predict ncRPIs by using deep learning conjoint with RF classifier.[44] More specifically, RNA sequences are first converted into *k*-mers sparse matrix,[40] which retains almost all amino acid compositions and order information. Then the singular value decomposition (SVD) is used to extract the feature vector for each sequence.[45] For protein sequences, a pseudo-Zernike moment (PZM) descriptor is used to extract the evolutionary information from the position-specific scoring matrix (PSSM).[42,46] Then, the stacked auto-encoder is further employed to automatically learn hidden high-level features from above mentioned features.[47] Finally, these reprehensive features are fed into RF classifiers to predict RPIs. To further improve the robustness and accuracy of our method, extra layers are employed to integrate different predictors. In the experimental, the proposed method was evaluated on three benchmark datasets including RPI488,[48] RPI1807,[20] and RPI2241[21] and compared with other state-of-the-art methods, such as lncPro,[25] RPISeq-RF,[21] RPI-Pred,[20] and IPMiner.[48] The experimental results showed that our method can achieve much better prediction performance on above datasets.

RESULTS AND DISCUSSION

In this study, we propose a deep learning method named RPI-SAN, which conjoins the stacked auto-encoder network (SAN) with RF classifiers and used PSSM with the Zernike moment and *k*-mers sparse matrix with SVD to predict the interactions of ncRNA-protein. First, we evaluate its predictive ability of RPIs on the RPI2241 dataset. Furthermore, we compare RPI-SAN with other state-of-the-art methods on different datasets to demonstrate the effectiveness and robustness of our approach. Then we predict ncRPIs on different datasets by using the trained model. Furthermore, we made a case study that shows, with specific examples, how RPI-SAN advanced studies regarding potential RPIs. Finally, we summarize, analyze, and discuss our method.

Evaluation of RPI-SAN's Capability to Predict RPIs

We first test our RPI-SAN approach to evaluate its capability to predict RPIs on the RPI2241 dataset. The details listed in the Tables S2 and S3 are as follows.

The mean accuracy of 5-fold cross-validation is 90.77%, the mean sensitivity is 86.17%, the mean specificity is 97.37%, the mean precision is 84.05%, and the Matthews correlation coefficient (MCC) is 82.27%. Their respective SDs are 0.52%, 0.81%, 1.71%, 1.26%, and 1.25%. Table S2 shows the 5-fold cross-validation details performed by RPI-SAN on the RPI2241 dataset, with the area under the receiver operating characteristic curve (AUC) achieving 0.962 as shown in Figure 1. Our method has achieved the best performance on the RPI2241 dataset in all methods.

Our method is manifested by three stacked separate predictors, a stacked auto-encoder with fine-tuning (SA-FT-RF), a stacked auto-encoder with RF (SA-RF), and RPISeq with RF (RPISeq-RF), with each individual predictor performing different effects on different data. The stacked auto-encoder performs well in accuracy and specificity, while RPISeq-RF specializes in precision and sensitivity. It is explained that individual predictors have weaker adaptability. It is necessary to integrate them together to give play to each other's strengths.

On the RPI2241 dataset, our RPI-SAN method performs much better than other predictors. Shown in Table S3, RPI-SAN performs at an accuracy of 90.77%, sensitivity of 86.17%, specificity of 97.37%, precision of 84.05%, MCC of 82.17%, and AUC of 0.962. It's the best model in these four contrasting predictors. SA-RF performs at an accuracy of 63.71%, sensitivity of 64.75%, specificity of 61.72%, precision of 65.74%, and MCC of only 27.49%. The accuracy, sensitivity, specificity, precision, and MCC of RPISeq-RF are 63.96%, 64.83%, 62.59%, 65.37%, and 27.98%, and those of SA-FT-RF are 90.52%, 87.71%, 94.78%, 86.18%, and 81.56%. lncPro performs at an accuracy of 65.4%, sensitivity of 65.9%, specificity of 64.0%, precision of 66.9%, and MCC of 31.0%, respectively. lncPro performs a little worse than RPI-SAN. It has some disadvantages; it can only predict protein sequences longer than 30, which fails in predicting shorter protein

Table 1. Comparing RPI-SAN with Other Methods on RPI4888, RPI1807 and RPI2241 Datasets

Datasets	Methods	Accuracy (%)	Sensitivity (%)	Specificity (%)	Precision (%)	MCC (%)	AUC
RPI488	IPMiner	89.1	93.9	83.1	94.5	78.4	0.914
	RPISeq-RF	88.0	92.6	82.2	93.2	76.2	0.903
	lncPro	87.0	90.0	82.7	91.0	74.0	0.901
	RPI-SAN	89.7[a]	94.3[a]	83.7	95.2[a]	79.3[a]	0.920[a]
RPI1807	RPI-Pred	93.0	95.0	N/A	94.0	N/A	0.97
	IPMiner	98.6[a]	98.2[a]	99.3	97.8[a]	97.2[a]	0.998
	RPISeq-RF	97.3	96.8	98.4	96.0	94.6	0.0996
	lncPro	96.9	96.5	98.1	95.5	93.8	0.994
	RPI-SAN	96.1	93.6	99.9	91.4	92.4	0.999[a]
RPI2241	RPI-Pred	84.0	78.0	N/A	88.0[a]	N/A	0.89
	IPMiner	82.4	83.3	81.2	83.6	65.0	0.906
	RPISeq-RF	63.96	64.83	62.59	65.37	27.98	0.690
	lncPro	65.4	65.9	64.0	66.9	31.0	0.722
	RPI-SAN	90.77[a]	86.17[a]	97.37[a]	84.05	82.27[a]	0.962[a]

[a]This measure of performance is the best among the compared methods for the individual dataset.

sequences. Since using RNAsubopt to predict RNA structure takes a long time, especially for long sequences, it only processes the first 4,095 nucleotides if the RNA sequence is longer than 4,095. These are the reasons why our method does not include lncPro in our stacked predictors.

Comparison between Different Assembling Strategies

In our RPI-SAN method, we use stacked assembling to integrate different classifiers. This time, we compare it with other general methods, such as majority voting and averaging. As the results show in Figure 1, stacked assembling attains an AUC of 0.962 on the RPI2241 dataset, which is better than the average method and each individual classifier. Logistic regression gets different weights for the stacked auto-encoder, stacked auto-encoder with fine-tuning, and RPISeq-RF by using the raw sequence feature, which is more robust and flexible than the average stacked auto-encoder.

Different predictors play different roles in the production of the final result. Stacked assembling improves the final prediction effect at different ranges. On the RPI488 and RPI1807 datasets, the three predictors have outputs similar to the RPI2241 dataset, which means a stronger correlation. So stacked assembling improves the AUC on RPI488 and RPI1807, but smaller than the improvement on RPI2241, which has a lower correlation. As a result, the stacked assembling is really effective for improving the final performance. So it is more significant on datasets with lower correlation.

Comparison with Other Methods

In order to verify the effectiveness and robustness of RPI-SAN, we compare it with other state-of-the-art methods in the same datasets. Here we have selected the RPI-Pred from the study by Suresh et al.[20] and the RPISeq-RF from the study by Muppirala et al.[21] because the RPISeq-RF performs better than RPISeq-SVM in this study. We have also selected the IPMiner from the study by Pan et al.[48] and the lncPro from the study by Lu et al.[25] Since these methods are not evaluated on the same criteria, we only compare the results of the same evaluation methods on the same datasets.

As shown in Table 1 and Figure S1, on the RPI488 dataset, our method performs a little better than any other method, with an accuracy of 89.7%, sensitivity of 94.3%, specificity of 83.7%, precision of 95.2%, MCC of 79.3%, and AUC of 0.92. The performance of each parameter is optimal. For the RPI1807 dataset, all methods except RPI-Pred give great performances with the accuracy and AUC greater than 95% (shown in Figure S2). Our method also gives a great performance. Although the accuracy is not best, it still attains a high accuracy of 96%. In terms of specificity and the important parameter AUC, our method is outstanding, achieving an AUC of 0.999. For the RPI2241 dataset, before our proposed method RPI-SAN, most methods did not work very well, especially in terms of accuracy, MCC, and AUC. Compared with the best methods already published, RPI-SAN improved the accuracy by almost 7%, specificity by more than 16%, MCC by over 17%, and AUC by more than 6%, respectively.

Predicting ncRPIs Using RPI-SAN

To further validate the ability of RPI-SAN to predict the interactions between ncRNA and protein, we use the RPI488 dataset to train the deep learning model and verify it on the NPInter v2.0 dataset.[49] There is no overlap between the two datasets. There are 10,412 interaction pairs in the NPInter v2.0, which can be divided into six organisms, and we conduct experiments on them separately. The results are shown in Table 2. RPI-SAN predicts the correct number of pairs of interactions on *Homo sapiens*, *Caenorhabditis elegans*, *Drosophila melanogaster*, *Saccharomyces cerevisiae*, *Mus musculus*, and *Escherichia*

Table 2. Predicted Performance of the RPI488 Trained Model on NPInter v2.0 Dataset

Organism	Number of Interaction Pairs	Predicted Number of Interaction Pairs	Accuracy (%)
Homo sapiens	6,975	6,928	99.33
Caenorhabditis elegans	36	29	80.56
Drosophila melanogaster	91	90	98.90
Saccharomyces cerevisiae	910	897	98.56
Mus musculus	2,198	2,153	97.95
Escherichia coli	202	177	87.62
Total	10,412	10,274	98.67

coli for 6,928, 29, 90, 897, 2,153, and 177, with an accuracy of 99.33%, 80.56%, 98.90%, 98.56%, 97.95%, and 87.62%, respectively. We finally predict that the correct number of ncRNA-protein pairs is 10,274, with a total accuracy of 98.67% on the independent dataset NPInter v2.0.

Table 3. Confirmed RNA-Protein Interactions with High Ranks in the Dataset of *Homo sapiens*

Protein ID	RNA ID	Probability
HNRNPA1	EPB41	0.867
TARDBP	CFTR	0.866
MBNL1	DMPK	0.863
PTBP1	CD40LG	0.859
SRP19	RN7SL1	0.857
SRSF1	TNNT2	0.856
ELAVL4	MYCN	0.853
ELAVL2	ID1	0.851
HNRNPC	CSF2	0.848
HNRNPD	ADRB1	0.847
EIF5A	RNU6-1	0.845
HNRNPD	AGTR1	0.842
ELAVL3	VEGFA	0.838
YBX1	CSF2	0.833
ZBP1	ACTB	0.831

Case Study: Potential RPIs of the Top-15 Ranks Verified from Database

After evaluating the effectiveness and robustness of the proposed model, we calculate the possibility of interaction for potential RNA-protein pairs in the dataset of *Homo sapiens*. The training data do not overlap with the testing data. The predicted RNA-protein pairs with high probability are considered as potential interacted pairs and further verified by Gene Ontology.[50] As a result, shown in Table 3, 15 interacted RNA-protein pairs are finally confirmed. Note that the high-ranked interactions that are not reported yet may also exist in reality. Based on these results, we anticipate that the proposed model is feasible to predict new RPIs.

Conclusions

In this study, we have proposed the computational method RPI-SAN based on deep learning with efficient features and stacked assembling to predict RPIs. We use PSSM and *k*-mers sparse matrix to extract efficient features from proteins and RNAs, respectively. Then such features will be fed into the SAN with RF predictors. The presented method gives a high performance with an accuracy of 90.77%, MCC of 82.27%, and an excellent AUC of 96.2% on the RPI2241 data-set. RPI-SAN also performs well on other previous popular datasets. Experimental results prove that the stacked auto-encoder can learn high-level features automatically from raw information, which is important for designing machine learning models. RPI-SAN gives a great performance on both RNA-protein and ncRPI prediction, which can prove that RPI-SAN is better than other state-of-the-art methods in some aspects. Through experiments, we also find that RPI-SAN has a better effect on large-scale datasets than small data-sets, which we will keep studying in further work. We researched the computational techniques for predicting the interaction of ncRNA-proteins because it is more convenient and rapid than traditional hand-tuning experiments and can accurately predict the potential ncRNA-protein interacted pairs, which provides reliable guidance for the further biological researches.

MATERIALS AND METHODS

Construction of Datasets

To evaluate the effectiveness and robustness of our approach, we conducted experiments on four different benchmark datasets, including RPI488, RPI1807, RPI2241, and NPInter v2.0.[49] The RPI488 is a non-redundant lncRPI dataset based on structure complexes,[51,52] which contains 488 lncRNA-protein pairs, including 245 non-interacting pairs and 243 interacting pairs. Here it is smaller than other RNA-protein datasets, with only 243 lncRPIs. The reason is that there are much fewer lncRNA-protein complexes in the Protein Data Bank (PDB)[53] database, where the ncRNA-protein complexes are downloaded from.[54] The dataset RPI1807 contains 1,807 positive ncRPI pairs, including 1,078 RNA chains and 1,807 protein chains. The number of negative ncRPI pairs is 1,436, which contain 493 RNA chains and 1,436 protein chains. It is established by parsing the Nucleic Acid Database (NAD), which provides the RNA-protein complex data and protein-RNA interface data. The RPI2241 dataset is constructed in a similar way and contains 2,241 interacting RNA-protein pairs. The NPInter v2.0 is an ncRPI from a non-structure-based source, containing 10,412 ncRNA-protein pairs and 449 chains of protein and 4,636 chains of ncRNA. Table 4 shows the details of the datasets used in this study.

Representation of the ncRNA and Protein Sequences

To obtain high effective features for deep learning models, each ncRNA-protein pair is represented as 486-feature vectors, in which 256 features are used to encode the RNA sequence, and 240 features are used to encode the protein sequence. RNAs are encoded by using the *k*-mers sparse matrix previously proposed in Zhu-Hong et al.[40] In

Table 4. The Details of the ncRNA-Protein Interaction Datasets

Dataset	Interaction Pairs	Number of Proteins	Number of RNAs
RPI488	243	25	247
RPI1807	1,807	1,807	1,078
RPI2241	2,241	2,043	332
NPInter v2.0	10,412	449	4,636

RPI488 is lncRNA-protein interactions based on structure complexes. PI369, RPI2241, and RPI1807 are RNA-protein interactions. NPInter2.0 and RPI13254 are ncRNA-protein interactions from non-structure-based source.

this method, we scan each RNA sequence (A, C, G, U) from left to right, stepping one nucleotide at a time, which is considered the characteristic of each nucleotide. Its k-1 consecutive nucleotides and k consecutive nucleotides are regarded as a unit. For any above-mentioned RNA sequences of length L, there would be 4^k different possible k-mers and $L-k+1$ k-mers appearing in the RNA sequence.

Each input of the RNA sequence is processed into a $4^k \times (L-k+1)$ k-mers sparse matrix R. When $R_jR_{j+1}R_{j+2}R_{j+3}$ are just equal to the ith k-mers among 4^k different k-mers, set the element $a_{ij}=1$. The rest can be dealt with in the same way. Then an input RNA sequence is converted into a $4^k \times (L-k+1)$ matrix R. In this study, the value of k is set to 4 to process the RNA sequence, which can be obtained from Table S1.

$$M = \left(a_{ij}\right)_{4^k} \times (L-k+1) \tag{1}$$

$$a_{ij} = \begin{cases} 1, & \text{if } R_jR_{j+1}R_{j+2}R_{j+3} = k-mer(i) \\ 0, & else \end{cases} \tag{2}$$

The 4-mer sparse matrix R is a low-rank matrix, while almost all of the information is retained, including sequence (AAAA, AAAC …UUUU) frequency, position, and order-hidden information in a protein sequence. Then, we use SVD to process a matrix R into a 1×256 vector feature.

Considering that RNA and protein sequences have different structures for protein amino acids sequences, we use a more biological method, the PSSM, to transform it. The PSSM algorithm containing biological evolution information was first used to detect distantly related protein, achieving great success in the prediction of the protein secondary structure and the protein binding site and the disordered regions prediction. The structure of PSSM is a $L \times 20$ matrix, while L rests with the length of the input protein sequence and 20 represents the number of naive amino acids. Supposing $p = \{b_{(i,j)}, i = 1,2,\ldots N \text{ and } j = 1,2,\ldots 20\}$, PSSM is represented as follows

$$P = \begin{bmatrix} b_{1,1} & \cdots & b_{1,20} \\ \vdots & \ddots & \vdots \\ b_{N,1} & \cdots & b_{N,20} \end{bmatrix}, \tag{3}$$

where $b_{i,j}$ in the i row of PSSM represents the probability of the ith residue being mutated into type j of 20 native amino acids during the procession of evolutionary in the protein from multiple sequence alignments. In experiments, we used the position-specific iterated BLAST (PSI-BLAST) tool to convert protein raw sequence into PSSM. We set the PSI-BLAST tool against the database of *SwissProt*, the number of iteration as 3, and err-value to 0.001, to get the best results. Both PSI-BLAST applications and the *SwissProt* database can be freely downloaded from http://blast.ncbi.nlm.nih.gov/Blast.cgi.

Then we extracted the PZM[41] features from the PSSM. PZM is widely used in the field of image processing and has achieved good results, which can extract features from the matrix more robustly and has less information redundancy. We set the PZM required parameter n, m = 30. Finally, a feature vector is obtained for each protein sequence.

SAN

Deep learning as a powerful vehicle has been widely used in different areas[19,22,23,43,55,56] and has received great attention in the field of ncRPI prediction.[57] Among these several deep-learning architectures, the SAN is more appropriate to our demand. The stacked auto-encoder has almost all the advantages of the deep neural network (DNN) and has an outstanding expressive ability. It is usually able to obtain the "hierarchical grouping" and "partial-global decomposition" features of the raw data. Since the stacked auto-encoder tends to be able to effectively represent the original input data, we use auto-encoder as a component element of a DNN with multiple layers.[44,55,58]

The SAN is composed of a multilayer neural network sparse auto-encoder and the output from the previous layer as input of the next layer as shown in Figure 2. With hyper parameter optimization, we get the best parameters of the stacked auto-encoder neural network. The sparse auto-encoder network is constructed like Figure 3. Error represents the error between the reconstructed data and the input, while the sparsity penalty stands for regularity limit for $L1$, which constrains the majority of each layer's node, which is 0, with only a few that are not 0.

Where the input x is in the form of d-dimension and the auto-encoder network maps X into the output $h(X)$:

$$h_{(w,b)}(x) = f\left(W^TX\right) = f\left(\sum_{i=1}^{n} w_ix_i + b\right), \tag{4}$$

where the f is activation function. When we select Sigmoid as the activation function,

$$f(z) = \frac{1}{1+e^{-z}}, \tag{5}$$

then the loss function is as follows:

$$L(X,W) = \|Wh - X\|^2 + \lambda\sum_j |h_j| . \tag{6}$$

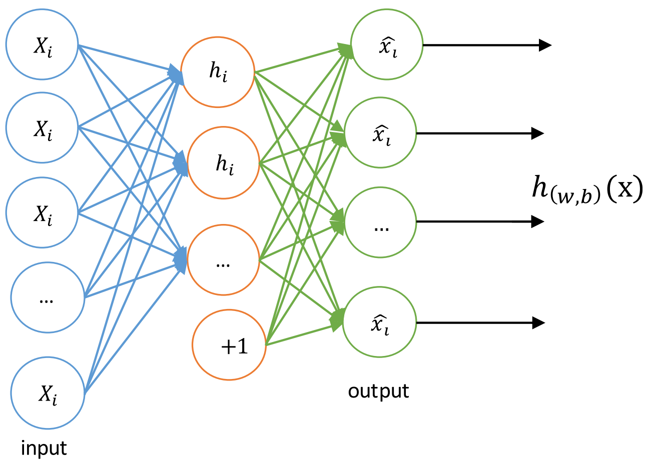

Figure 2. The Construction of Stacked Auto-Encoder Network

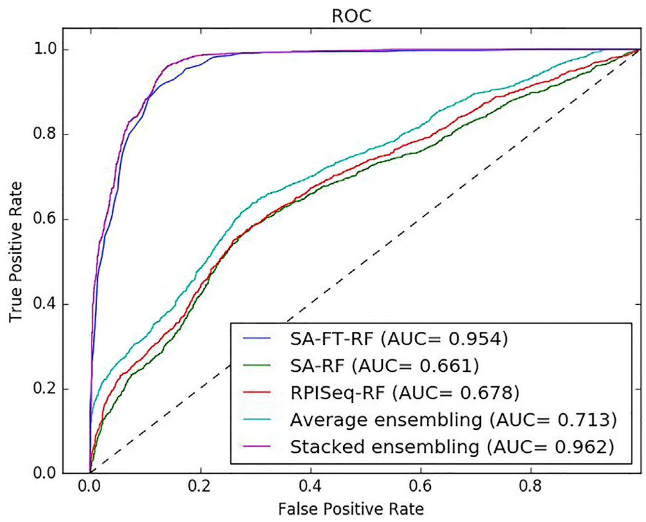

Figure 3. The Construction of Sparse Auto-Encoder

Usually, each layer of neural network includes a certain number of neurons. Then, the multilayer neural network makes up a stacked network of sequential connected layers, while the output of the previous layers is the input of the next layers:

$$a^{(l)} = f\left(z^{(l)}\right) \tag{7}$$

$$z^{(l+1)} = W^{(l,1)}a^{(l)} + b^{(l,1)}. \tag{8}$$

Among them, $a^{(n)}$ is the activation value of the deepest hidden unit, which is a higher order representation of the input value. By using $a^{(n)}$ as the input feature of the softmax classifier, the features learned in the deep auto-encoder network can be used for classification problems. We use the stochastic gradient descent (SGD)[59] to optimize the reconstruction error between X and z, which can be measured by using the squared error.

Stacking multiple auto-encoders[47] consists of a stacked auto-encoder, a DNN that can learn high-level features automatically.[60,61] To get a better performance, we use greedy layer-wise learning, which can train each layer individually to optimize objective functions when learning the stacked auto-encoder parameters. In our network, we use two types of layers: full-connected and dropout layers.[62] For the dropout layer, it set some node activations to 0 with a certain probability to avoid overfitting for model training. We also add an extra soft-max layer for fine tuning, with the ReLu function as activation for the outputs from the conjoined multiple- layer network of RNA and protein as the last hidden layer, which is trained by using real label information to update weights and bias parameters for SAN.[63,64] Then we use SGD (with different learn rates and momentums for different datasets) to minimize cross entropy loss function and Adam to minimize mean squared error for each de-noising auto-encoder layer, and the dropout probability is set to 0.5 during the model training.[65,66] In this study, we use the keras library to implement the stacked auto-encoder and set the parameters *batch_size* and *nb_epoch* to 100, respectively. The details about keras can be found at http://github.com/fchollet/keras.

Stacked Assembling

Ordinarily, different classifiers have different performances in different datasets. In fact, there is no single classifier that can be adapted to all kinds of datasets. An extra-stacked assembling layer is used in our deep learning network to integrate the individual multiple classifier outputs to gain the approximate optimal target function. Previous works have proposed majority voting[36] and average individual classifiers outputs.[67]

In our study, using multiple layer neural networks following the deep learning intuition, we define the operating mechanism as the level 0 classifiers' outputs that will be fed into the level 1 classifier as training data. Where level 0 is the original layer and level 1 the next sequential layer, how to obtain the outputs from separate classifiers will be worked out. In our network, the outputs of the level 0 layer classifiers are the predicted probability score, while the successive level 1 classifier is logistic regression. When the weight of logistic regression for each individual classifier is the same, it degenerates to average treatment. When only one weight is not zero, it is more like a majority voting method:

$$P_w(\pm 1|p) = \frac{1}{1 + e^{-w^T p(\pm 1|p)}}, \tag{9}$$

where p is the probability score vector outputs of the individual classifiers and w is the weight vector for every single different classifier. The logistic regression is from Scikit-learn.[68]

Performance Evaluation

In this study, we trained the deep learning model to classify whether ncRNA and protein interact with each other or not. The 5-fold cross-validation method is used to evaluate the performance of our study, which randomly divides all the datasets into five equal parts. In each validation, one of them is taken as the testing set, and the other four parts are taken as the training set. The testing and training data

do not overlap with each other to guarantee the unprejudiced comparison. We take the average and SDs of these results as the final validation result. We follow the widely used evaluation measure to evaluate our method, including accuracy (Acc.), sensitivity (Sen.), specificity (Spec.), precision (Prec.), and MCC defined as:

$$\text{Acc.} = \frac{TN + TP}{TN + TP + FN + FP} \tag{10}$$

$$\text{Sen.} = \frac{TP}{TP + FN} \tag{11}$$

$$\text{Spec.} = \frac{TN}{TN + FP} \tag{12}$$

$$\text{Prec.} = \frac{TP}{TP + FP} \tag{13}$$

$$\text{MCC} = \frac{TP \times TN - FP \times FN}{\sqrt{(TP + FP)(TP + FN)(TN + FP)(TN + FN)}}, \tag{14}$$

where TN indicates the correctly predicted negative number, TP denotes the correctly predicted positive number, FN represents the wrongly predicted negative number, and FP stands for the wrongly predicted positive number. Certainly, the receiver operating characteristic (ROC) curve and the area under ROC curve (AUC) are also exploited to evaluate the performance of classifiers.

SUPPLEMENTAL INFORMATION

Supplemental Information includes two figures and three tables and can be found with this article online at https://doi.org/10.1016/j.omtn.2018.03.001.

AUTHOR CONTRIBUTIONS

H-C.Y. and Z-H.Y. conceived the algorithm, carried out analyses, prepared the data sets, carried out experiments, and wrote the manuscript. D-S.H., X.L., T-H.J., and L-P.L. wrote the manuscript and analyzed experiments. All authors read and approved the final manuscript.

CONFLICTS OF INTEREST

The authors declare no conflict of interest.

ACKNOWLEDGMENTS

This work was supported in part by the National Natural Science Foundation of China under grants 61732012, 61520106006, and 61722212 and in part by the Pioneer Hundred Talents Program of Chinese Academy of Sciences.

REFERENCES

1. Taft, R.J., Pheasant, M., and Mattick, J.S. (2007). The relationship between non-protein-coding DNA and eukaryotic complexity. BioEssays *29*, 288–299.
2. Esteller, M. (2011). Non-coding RNAs in human disease. Nat. Rev. Genet. *12*, 861–874.
3. Li, J.H., Liu, S., Zhou, H., Qu, L.H., and Yang, J.H. (2014). starBase v2.0: decoding miRNA-ceRNA, miRNA-ncRNA and protein-RNA interaction networks from large-scale CLIP-Seq data. Nucleic Acids Res. *42*, D92–D97.
4. Djebali, S., Davis, C.A., Merkel, A., Dobin, A., Lassmann, T., Mortazavi, A., Tanzer, A., Lagarde, J., Lin, W., Schlesinger, F., et al. (2012). Landscape of transcription in human cells. Nature *489*, 101–108.
5. Consortium, T.E.P.; ENCODE Project Consortium (2012). An integrated encyclopedia of DNA elements in the human genome. Nature *489*, 57–74.
6. Derrien, T., Johnson, R., Bussotti, G., Tanzer, A., Djebali, S., Tilgner, H., Guernec, G., Martin, D., Merkel, A., Knowles, D.G., et al. (2012). The GENCODE v7 catalog of human long noncoding RNAs: analysis of their gene structure, evolution, and expression. Genome Res. *22*, 1775–1789.
7. Harrow, J., Frankish, A., Gonzalez, J.M., Tapanari, E., Diekhans, M., Kokocinski, F., Aken, B.L., Barrell, D., Zadissa, A., Searle, S., et al. (2012). GENCODE: the reference human genome annotation for The ENCODE Project. Genome Res. *22*, 1760–1774.
8. Brown, C.J., Ballabio, A., Rupert, J.L., Lafreniere, R.G., Grompe, M., Tonlorenzi, R., and Willard, H.F. (1991). A gene from the region of the human X inactivation centre is expressed exclusively from the inactive X chromosome. Nature *349*, 38–44.
9. Lee, J.T., Davidow, L.S., and Warshawsky, D. (1999). Tsix, a gene antisense to Xist at the X-inactivation centre. Nat. Genet. *21*, 400–404.
10. Brannan, C.I., Dees, E.C., Ingram, R.S., and Tilghman, S.M. (1990). The product of the H19 gene may function as an RNA. Mol. Cell. Biol. *10*, 28–36.
11. Sleutels, F., Zwart, R., and Barlow, D.P. (2002). The non-coding Air RNA is required for silencing autosomal imprinted genes. Nature *415*, 810–813.
12. Rinn, J.L., Kertesz, M., Wang, J.K., Squazzo, S.L., Xu, X., Brugmann, S.A., Goodnough, L.H., Helms, J.A., Farnham, P.J., Segal, E., and Chang, H.Y. (2007). Functional demarcation of active and silent chromatin domains in human HOX loci by noncoding RNAs. Cell *129*, 1311–1323.
13. Kretz, M., Siprashvili, Z., Chu, C., Webster, D.E., Zehnder, A., Qu, K., Lee, C.S., Flockhart, R.J., Groff, A.F., Chow, J., et al. (2013). Control of somatic tissue differentiation by the long non-coding RNA TINCR. Nature *493*, 231–235.
14. Khorshid, M., Rodak, C., and Zavolan, M. (2011). CLIPZ: a database and analysis environment for experimentally determined binding sites of RNA-binding proteins. Nucleic Acids Res. *39*, D245–D252.
15. Huang, Y.A., Chan, K., and You, Z.H. (2018). Constructing prediction models from expression profiles for large scale lncRNA-miRNA interaction profiling. Bioinformatics *34*, 812–819.
16. Li, Z., Han, P., You, Z.H., Li, X., Zhang, Y., Yu, H., Nie, R., and Chen, X. (2017). In silico prediction of drug-target interaction networks based on drug chemical structure and protein sequences. Sci. Rep. *7*, 11174.
17. Ray, D., Kazan, H., Chan, E.T., Peña Castillo, L., Chaudhry, S., Talukder, S., Blencowe, B.J., Morris, Q., and Hughes, T.R. (2009). Rapid and systematic analysis of the RNA recognition specificities of RNA-binding proteins. Nat. Biotechnol. *27*, 667–670.
18. Licatalosi, D.D., Mele, A., Fak, J.J., Ule, J., Kayikci, M., Chi, S.W., Clark, T.A., Schweitzer, A.C., Blume, J.E., Wang, X., et al. (2008). HITS-CLIP yields genome-wide insights into brain alternative RNA processing. Nature *456*, 464–469.
19. Alipanahi, B., Delong, A., Weirauch, M.T., and Frey, B.J. (2015). Predicting the sequence specificities of DNA- and RNA-binding proteins by deep learning. Nat. Biotechnol. *33*, 831–838.
20. Suresh, V., Liu, L., Adjeroh, D., and Zhou, X. (2015). RPI-Pred: predicting ncRNA-protein interaction using sequence and structural information. Nucleic Acids Res. *43*, 1370–1379.
21. Muppirala, U.K., Honavar, V.G., and Dobbs, D. (2011). Predicting RNA-protein interactions using only sequence information. BMC Bioinformatics *12*, 489.
22. Liu, B., Fang, L., Long, R., Lan, X., and Chou, K.C. (2016). iEnhancer-2L: a two-layer predictor for identifying enhancers and their strength by pseudo k-tuple nucleotide composition. Bioinformatics *32*, 362–369.
23. Liu, B., Yang, F., Huang, D.S., and Chou, K.C. (2017). iPromoter-2L: a two-layer predictor for identifying promoters and their types by multi-window-based PseKNC. Bioinformatics *34*, 33–40.

24. Liu, B., Zhang, D., Xu, R., Xu, J., Wang, X., Chen, Q., Dong, Q., and Chou, K.C. (2014). Combining evolutionary information extracted from frequency profiles with sequence-based kernels for protein remote homology detection. Bioinformatics *30*, 472–479.
25. Lu, Q., Ren, S., Lu, M., Zhang, Y., Zhu, D., Zhang, X., and Li, T. (2013). Computational prediction of associations between long non-coding RNAs and proteins. BMC Genomics *14*, 651.
26. Bellucci, M., Agostini, F., Masin, M., and Tartaglia, G.G. (2011). Predicting protein associations with long noncoding RNAs. Nat. Methods *8*, 444–445.
27. Agostini, F., Zanzoni, A., Klus, P., Marchese, D., Cirillo, D., and Tartaglia, G.G. (2013). catRAPID omics: a web server for large-scale prediction of protein-RNA interactions. Bioinformatics *29*, 2928–2930.
28. Livi, C.M., and Blanzieri, E. (2014). Protein-specific prediction of mRNA binding using RNA sequences, binding motifs and predicted secondary structures. BMC Bioinformatics *15*, 123.
29. Wang, Y., You, Z., Li, X., Chen, X., Jiang, T., and Zhang, J. (2017). PCVMZM: Using the probabilistic classification vector machines model combined with a zernike moments descriptor to predict protein-protein interactions from protein sequences. Int. J. Mol. Sci. *18*, 1029.
30. Wang, Y.B., You, Z.H., Li, X., Jiang, T.H., Chen, X., Zhou, X., and Wang, L. (2017). Predicting protein-protein interactions from protein sequences by a stacked sparse autoencoder deep neural network. Mol. Biosyst. *13*, 1336–1344.
31. Li, J.Q., You, Z.H., Li, X., Ming, Z., and Chen, X. (2017). PSPEL: In silico prediction of self-interacting proteins from amino acids sequences using ensemble learning. IEEE/ACM Trans Comput Biol Bioinform *14*, 1165–1172.
32. Liu, B., Chen, J., and Wang, X. (2015). Application of learning to rank to protein remote homology detection. Bioinformatics *31*, 3492–3498.
33. Liu, B., Liu, F., Wang, X., Chen, J., Fang, L., and Chou, K.C. (2015). Pse-in-One: A web server for generating various modes of pseudo components of DNA, RNA, and protein sequences. Nucleic Acids Res *43* (Web Server issue), W65–W71.
34. You, Z.H., Huang, Z.A., Zhu, Z., Yan, G.Y., Li, Z.W., Wen, Z., and Chen, X. (2017). PBMDA: A novel and effective path-based computational model for miRNA-disease association prediction. PLoS Comput. Biol. *13*, e1005455.
35. Chen, X., Yan, C.C., Zhang, X., and You, Z.H. (2017). Long non-coding RNAs and complex diseases: from experimental results to computational models. Brief. Bioinform. *18*, 558–576.
36. Breiman, L. (2001). Random Forest. Mach. Learn. *45*, 5–32.
37. Vapnik, V.F. (1998). Statistical Learning Theory. In Encyclopedia of the Sciences of Learning 41.4, N.M. Seel, ed. (Springer), p. 3185.
38. Pancaldi, V., and Bähler, J. (2011). In silico characterization and prediction of global protein-mRNA interactions in yeast. Nucleic Acids Res. *39*, 5826–5836.
39. Shen, J., Zhang, J., Luo, X., Zhu, W., Yu, K., Chen, K., Li, Y., and Jiang, H. (2007). Predicting protein-protein interactions based only on sequences information. Proc. Natl. Acad. Sci. USA *104*, 4337–4341.
40. Zhu-Hong, Y., MengChu, Z., Xin, L., and Shuai, L. (2017). Highly efficient framework forpredicting interactions between proteins. IEEE Trans. Cybern. *47*, 731–743.
41. Haddadnia, J., Ahmadi, M., and Faez, K. (2003). An efficient feature extraction method with pseudo-zernike moment in RBF neural network-based human face recognition system. EURASIP J. Adv. Signal Process. *2003*, 1–12.
42. Ahmad, S., and Sarai, A. (2005). PSSM-based prediction of DNA binding sites in proteins. BMC Bioinformatics *6*, 33.
43. Maaloe, L., Arngren, M., and Winther, O. (2015). Deep belief nets for topic modeling. Comput. Sci.
44. LeCun, Y., Bengio, Y., and Hinton, G. (2015). Deep learning. Nature *521*, 436–444.
45. Lathauwer, L.D., Moor, B.D., and Vandewalle, J. (2000). A multilinear singular value decomposition. SIAM J. Matrix Anal. Appl. *21*, 1253–1278.
46. Jeong, J.C., Lin, X., and Chen, X.W. (2011). On position-specific scoring matrix for protein function prediction. IEEE/ACM Trans. Comput. Biol. Bioinformatics *8*, 308–315.
47. Vincent, P., Larochelle, H., Lajoie, I., Bengio, Y., and Manzagol, P.A. (2010). Stacked denoising autoencoders: learning useful representations in a deep network with a local denoising criterion. J. Mach. Learn. Res. *11*, 3371–3408.
48. Pan, X., Fan, Y.X., Yan, J., and Shen, H.B. (2016). IPMiner: hidden ncRNA-protein interaction sequential pattern mining with stacked autoencoder for accurate computational prediction. BMC Genomics *17*, 582.
49. Yuan, J., Wu, W., Xie, C., Zhao, G., Zhao, Y., and Chen, R. (2014). NPInter v2.0: an updated database of ncRNA interactions. Nucleic Acids Res. *42*, D104–D108.
50. Ashburner, M., Ball, C.A., Blake, J.A., Botstein, D., Butler, H., Cherry, J.M., Davis, A.P., Dolinski, K., Dwight, S.S., Eppig, J.T., et al.; The Gene Ontology Consortium (2000). Gene ontology: tool for the unification of biology. Nat. Genet. *25*, 25–29.
51. Huang, Y., Niu, B., Gao, Y., Fu, L., and Li, W. (2010). CD-HIT Suite: a web server for clustering and comparing biological sequences. Bioinformatics *26*, 680–682.
52. Lewis, B.A., Walia, R.R., Terribilini, M., Ferguson, J., Zheng, C., Honavar, V., and Dobbs, D. (2011). PRIDB: a Protein-RNA interface database. Nucleic Acids Res. *39*, D277–D282.
53. Berman, H.M., Westbrook, J., Feng, Z., Gilliland, G., Bhat, T.N., Weissig, H., Shindyalov, I.N., and Bourne, P.E. (2000). The Protein Data Bank. Nucleic Acids Res. *28*, 235–242.
54. Puton, T., Kozlowski, L., Tuszynska, I., Rother, K., and Bujnicki, J.M. (2012). Computational methods for prediction of protein-RNA interactions. J. Struct. Biol. *179*, 261–268.
55. Bengio, Y., Courville, A., and Vincent, P. (2013). Representation learning: a review and new perspectives. IEEE Trans. Pattern Anal. Mach. Intell. *35*, 1798–1828.
56. Zhou, J., and Troyanskaya, O.G. (2015). Predicting effects of noncoding variants with deep learning-based sequence model. Nat. Methods *12*, 931–934.
57. Cook, K.B., Hughes, T.R., and Morris, Q.D. (2015). High-throughput characterization of protein-RNA interactions. Brief. Funct. Genomics *14*, 74–89.
58. Hinton, G.E., and Salakhutdinov, R.R. (2006). Reducing the dimensionality of data with neural networks. Science *313*, 504–507.
59. Le, Q.V., Ranzato, M., Monga, R., Devin, M., Chen, K., Corrado, G.S., Dean, J., and Ng, A.Y. (2012). Building high-level features using large scale unsupervised learning. https://static.googleusercontent.com/media/research.google.com/en//pubs/archive/38115.pdf.
60. Ramsundar, B., Kearnes, S., Riley, P., Webster, D., Konerding, D., and Pande, V. (2015). Massively Multitask Networks for Drug Discovery. Comput. Sci.
61. McHugh, C.A., Russell, P., and Guttman, M. (2014). Methods for comprehensive experimental identification of RNA-protein interactions. Genome Biol. *15*, 203.
62. Srivastava, N., Hinton, G., Krizhevsky, A., Sutskever, I., and Salakhutdinov, R. (2014). Dropout: a simple way to prevent neural networks from overfitting. J. Mach. Learn. Res. *15*, 1929–1958.
63. Glorot, X., Bordes, A., and Bengio, Y. (2011). Deep Sparse Rectifier Neural Networks. Proceedings of the 14th International Conference on Artificial Intelligence and Statistics (AISTATS) *15*, 315–323.
64. Krizhevsky, A., Sutskever, I., and Hinton, G.E. (2012). ImageNet classification with deep convolutional neural networks. NIPS'12 Proceedings of the 25th International Conference on Neural Information Processing Systems *1*, 1097–1105.
65. Dahl, G.E., Sainath, T.N., and Hinton, G.E. (2013). Improving deep neural networks for LVCSR using rectified linear units and dropout. 1988 International Conference on Acoustics, Speech and Signal Processing *26*, 8609–8613.
66. Kingma, D.P., and Ba, J. (2014). Adam: a method for stochastic optimization. Comput. Sci.
67. Pan, X.Y., Tian, Y., Huang, Y., and Shen, H.B. (2011). Towards better accuracy for missing value estimation of epistatic miniarray profiling data by a novel ensemble approach. Genomics *97*, 257–264.
68. Pedregosa, F., Varoquaux, G., Gramfort, A., Michel, V., Thirion, B., Grisel, O., Blondel, M., Prettenhofer, P., Weiss, R., and Dubourg, V. (2013). Scikit-learn: machine learning in python. J. Mach. Learn. Res. *12*, 2825–2830.

OMTN, Volume *11*

Supplemental Information

A Deep Learning Framework for Robust and Accurate Prediction of ncRNA-Protein Interactions Using Evolutionary Information

Hai-Cheng Yi, Zhu-Hong You, De-Shuang Huang, Xiao Li, Tong-Hai Jiang, and Li-Ping Li

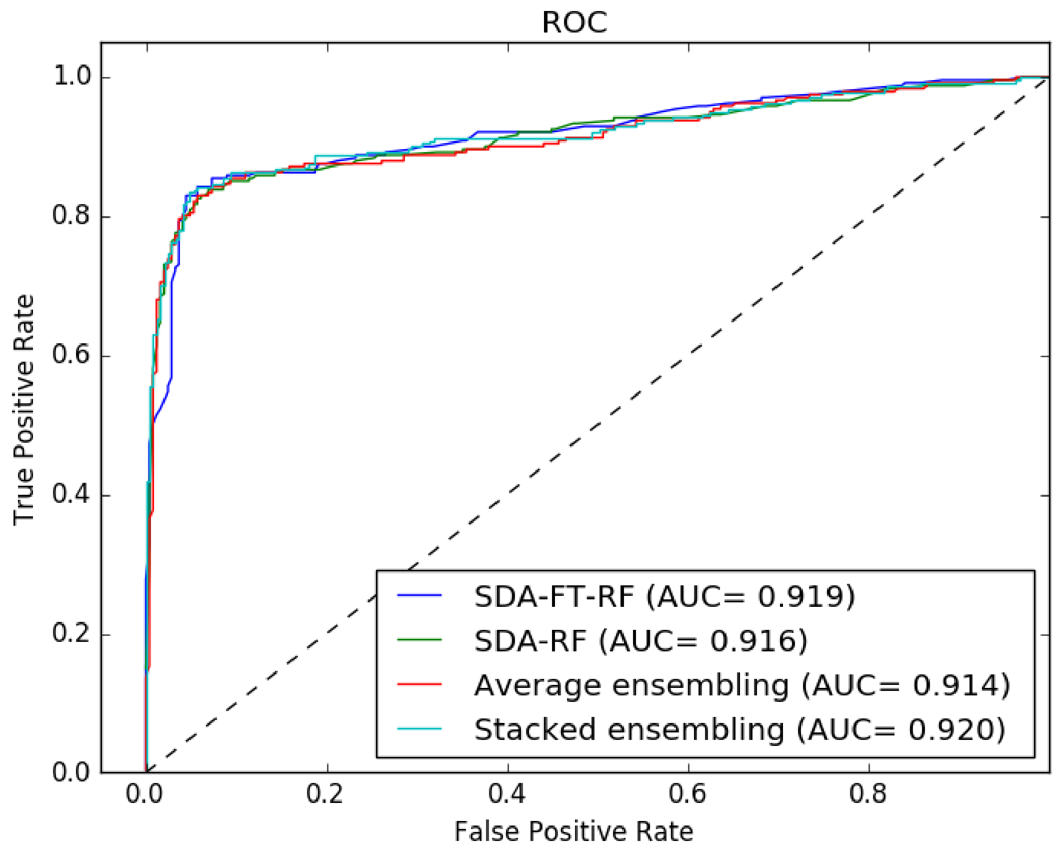

Figure S1: Performance on RPI488. Performance comparison between RPI-SAN, SDA-FT-RF, SDA-RF. Performance comparison between different assembling strategies.

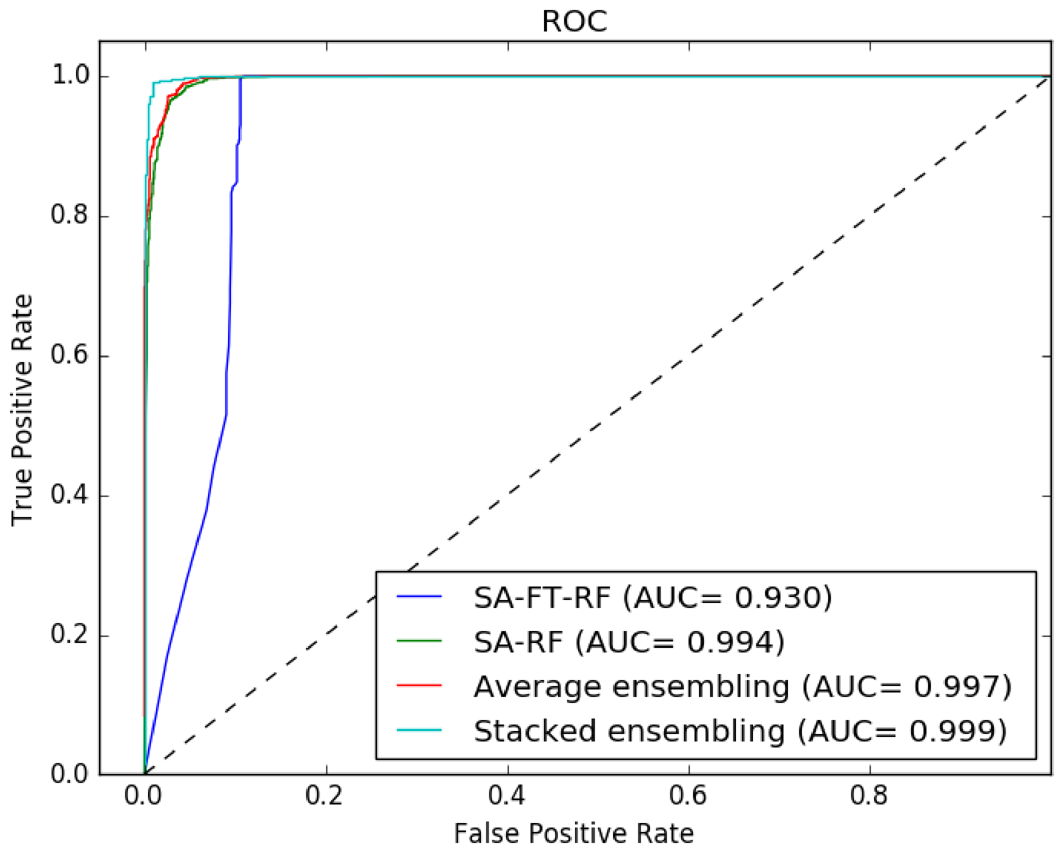

Figure S2: Performance on RPI1807. Performance comparison between RPI-SAN, SA-FT-RF, SA-RF. Performance comparison between different assembling strategies.

Table S1. K-mer sparse matrix based representation of RNA sequence.

	**$R_1R_2R_3R_4$**	**$R_2R_3R_4R_5$**	**…**	**$R_{L-3}R_{L-2}R_{L-1}R_L$**
**AAAA**	a_{11}	a_{12}	…	$a_{1,L-k+1}$
**AAAC**	a_{21}	a_{22}	…	$a_{2,L-k+1}$
**AACA**	a_{31}	a_{32}	…	$a_{3,L-k+1}$
**…**	…	…	…	…
**UUUU**	$a_{256,1}$	$a_{256,2}$	…	$a_{256,L-k+1}$

Table S2. The 5-fold cross-validation details on RPI2241 dataset.

Fold set	Accuracy(%)	Sensitivity(%)	Specificity(%)	Precision(%)	MCC(%)
**1**	90.50	86.08	96.82	84.06	81.63
**2**	90.50	85.38	97.96	82.91	81.89
**3**	91.65	85.80	99.99	83.14	84.46
**4**	90.84	87.53	95.46	86.11	82.00
**5**	90.38	86.06	96.60	84.03	81.36
**Average**	90.77±0.52	86.17±0.81	97.37±1.71	84.05±1.26	82.27±1.25

Table S3. Actual performance of individual predictors on RPI2241 dataset.

Predictors	Accuracy(%)	Sensitivity(%)	Specificity(%)	Precision(%)	MCC(%)
**SA-RF**	63.71	64.75	61.72	65.74	27.49
**SA-FT-RF**	90.52	**87.71**	94.78	**86.18**	81.56
**RPISeq-RF**	63.96	64.83	62.59	65.37	27.98
**lncPro**	65.4	65.9	64.0	66.9	31.0
**RPI-SAN**	**90.77**	86.17	**97.37**	84.05	**82.27**
